# Global research trends on RF-EMR and reproductive health: Bibliometric insights and mechanistic review

**DOI:** 10.1016/j.bbrep.2026.102832

**Published:** 2026-10-09

**Authors:** Rohit Gautam, Anuj Kumar Tomar, Shweta Tanwar, Poulami Mazumder, Anita Sharma, Asha Verma, Taruna Arora

**Affiliations:** a Division of Reproductive, Child Health and Nutrition, Indian Council of Medical Research, New Delhi, India; b School of Environmental Sciences, Jawaharlal Nehru University, New Delhi, India; c Indian Council of Medical Research, New Delhi, India; d Department of Reproductive Medicine and Surgery, Sawai Man Singh Medical College, Jaipur, Rajasthan, India

**Keywords:** Mobile phone radiation, Reproductive health, RF-EMR, Oxidative stress, Bibliometric analysis

## Abstract

The widespread adoption of mobile phones has raised public health concerns regarding the biological effects of radiofrequency electromagnetic radiation (RF-EMR), with reproductive health considered a potentially vulnerable domain. This study systematically characterized the global research landscape through a combined bibliometric analysis and mechanistic narrative review. Web of Science Core Collection was searched on 19 February 2025 with no year restriction. After applying inclusion and exclusion criteria, 828 publications were retained. Bibliometric networks were constructed using VOSviewer (version 1.6.20) at institutional, country, author, keyword, and journal levels. A complementary mechanistic review was conducted independently of the bibliometric dataset. Publication output increased substantially from 2005, peaking between 2018 and 2020. The United States and China were the most frequently represented countries (152 and 151 documents, respectively). Keyword co-occurrence identified oxidative stress as the dominant mechanistic theme (110 occurrences; TLS = 838), co-occurring with apoptosis, sperm quality, DNA fragmentation, testosterone, and spermatogenesis. A temporal shift from genetic risk studies towards targeted mechanistic investigations was observed. Evidence from in vitro, animal, and human studies suggests RF-EMR may adversely affect reproductive health through oxidative stress, mitochondrial dysfunction, DNA damage, apoptosis, hormonal disruption, and impaired spermatogenesis, though direct extrapolation to human outcomes requires caution. This study maps global research trends in RF-EMR and reproductive health and identifies key collaborative networks and mechanistic themes. Standardized experimental designs, long-term human studies, and interdisciplinary approaches are needed to resolve outstanding uncertainties.

## Highlights

• Analyzed 828 publications to map global RF-EMR and reproductive health research trends.
• Oxidative stress emerged as the leading mechanism linked to reproductive toxicity.
• Research focuses on sperm quality, DNA damage, apoptosis, and hormonal disruption.
• Highlights evidence gaps and priorities for future reproductive health studies.

## 1 Introduction

Mobile phones have become ubiquitous in contemporary life. Over the past two decades, rapid technological advancements have transformed mobile phones from luxury devices into indispensable tools of modern life [1]. Mobile phone emits radiofrequency electromagnetic radiation (RF-EMR), which has raised concerns about potential health risk [2]. RF-EMR generated from mobile phones comes under non-ionizing radiation which does not have enough energy to break chemical bond. But long-term exposure can leads to biochemical and molecular changes [3,4].

Recent studies on humans and animal models have shown the effects of radio frequency radiation emitted from mobile phone and other sources on eyes, reproductive system, brain, liver and other organs [[5], [6], [7], [8]]. Findings from various epidemiological studies have also suggested that long term uses of radiofrequency radiation emitted from mobile phone and other wireless devices can affect reproductive system and nervous system of children and adults [[9], [10], [11]].

Infertility is defined as the inability to conceive after a year of unprotected intercourse [12]. Infertility can be caused due to multiple reasons, but infertility due to exposure to radiofrequency electromagnetic fields is a new area of concern. The metabolic health and reproductive potential of offspring can be influenced by father's reproductive health [13]. Studies have indicated that sperm quality is highly susceptible to lifestyle and environmental factors [14]. These factors can be age, sedentary lifestyle, alcohol, smoking, drug abuse, exposure to heavy metals, endocrine disrupters and radiofrequency electromagnetic radiation [[15], [16], [17]].

The International Agency for Research on Cancer (IARC), a part of World Health Organization classified RF-EMR as Group 2B - Possibly carcinogenic to human [18]. A significant concern arises from how we carry our phones in trouser pockets, placing them in close, anatomical proximity to our reproductive organs; this may result in disrupting spermatogenesis [19].

RF-EMF is non-ionizing radiation that can affect reproductive system in thermal and non-thermal ways [20]. Prior studies have revealed that RF-EMR affects biological system via generating eddy currents which disrupt membrane potential [21]. Additionally, numerous in vitro and in vivo studies have implicated that RF-EMR induces oxidative stress (generation of free radical) via disrupting mitochondrial activity, which affects cell viability and DNA integrity [[22], [23], [24]]. Sperm cell membrane is rich in poly unsaturated fatty acid so more prone to oxidative damage which affects sperm viability and motility [25]. Studies have also showed that RF-EMR emitted from mobile phone base station affects semen parameters in sub fertile men [26]. Further in recent animal studies exposure of female Wistar rat to 5G lower band (700 MHz) have resulted in significant increase in testosterone level, oxidative stress markers and no DNA damage [27]. In another study findings revealed that prolonged phone use and EMF exposure during pregnancy may increase the risk of CAKUT (Congenital anomalies of the kidney and urinary tract) in children [28].

Therefore, the present study aimed to systematically evaluate the global research landscape on mobile phone radiation and reproductive health through a bibliometric analysis combined with a review of recent experimental and epidemiological findings. Bibliometric analysis includes mapping publication trends, geographic and institutional productivity, collaboration networks, and the thematic evolution of research focus in this domain.

## 2 Materials and methods

### 2.1 Search strategy

A detailed literature search was done using the Web of Science database to find relevant publications evaluating the impact of mobile phone radiation on reproductive system. Web of Science was selected as the primary database because it provides comprehensive and structured bibliometric metadata including institutional affiliations, country designations, citation linkages, and journal classifications that are essential for accurate network visualization using VOSviewer. This database has also been widely adopted in prior bibliometric analyses in the fields of bioelectromagnetics and environmental health. The search was carried out on 19 February 2025 and no publication year restriction was applied; all records from database inception up to 19 February 2025 were eligible for inclusion.

A combination of MeSH terms and keywords related to mobile phone radiation such as “Mobile Phone Radiation”, “Cell Phone Radiation”, “Microwave Radiation”, “Radio Frequency Radiation”, “Non-Ionizing Radiation”, “RFR”, “1G”, “2G”, “3G”, “4G”, “5G”, “Wi-Fi”, “2.45 GHz”, “Mobile Phone Tower”, “Cell Phone Tower”, and “Telecommunication Tower” was used. To specifically capture literature reproductive health, terms such as “male infertility’, “male reproductive system”, “semen quality”, “sperm”, “spermatogenesis”, “testosterone”, “spermatozoa”, “testis”, “Leydig cells”, “Sertoli cells”, “Ovary”, “Ovary Function”, “Ovarian Reserve”, “Pregnancy Outcomes”, “Oogenesis”, “FSH”, “Follicle Stimulating Hormone”, “LH” and “Luteinizing Hormone” etc were included.

The search was performed across all fields to ensure comprehensive retrieval of relevant records. These two searches were then combined using the Boolean operator AND, yielding 877 records. The complete search string, including all field tags (TS = Topic Search), Boolean operators, and term combinations, is provided in Table S1. Inclusion criteria were: (1) original research articles or review articles; (2) published in English; (3) indexed in the WoS Core Collection. Exclusion criteria were: (1) conference proceedings, book chapters, editorials, and letters; (2) publications in languages other than English; (3) records with incomplete bibliometric metadata. After applying these filters, a total of 828 publications were identified and included for bibliometric analysis. Of the 877 records initially retrieved by combining search strings #1 and #2, 49 records were excluded through database-level filters applied directly within Web of Science. Exclusions were based on document type (retaining only original research articles and review articles; conference proceedings, editorials, letters, and corrections were excluded) and language (retaining only English-language publications). No manual title and abstract screening was performed, as the bibliometric analysis required complete and structured metadata available within the WoS Core Collection rather than content-based eligibility assessment. A final dataset of 828 publications was retained for analysis.

A final dataset of 828 publications was retained for analysis. It is important to note that the 828-record WoS dataset formed the basis for all bibliometric analyses (publication trends, country/institutional collaboration, author networks, keyword co-occurrence, and journal analysis). The mechanistic discussion presented in Section 4 draws additionally on targeted literature cited within those sections and is not limited to the bibliometric dataset.

### 2.2 Bibliometric tools

To identify research patterns, collaborations, and thematic trends within the field; retrieved data was exported and analyzed using bibliometric tool. This allowed in getting a comprehensive overview of the intellectual and collaboration structure of the research landscape. VOSviewer (version 1.6.20, Leiden University) was used for network construction and visualization. Co-authorship networks were constructed at the institutional, country, and author levels using a minimum threshold of three co-authored publications. Keyword co-occurrence networks were built using author keywords with a minimum occurrence threshold of five. Journal co-citation networks were constructed with a minimum co-citation threshold of five. In all networks, total link strength (TLS) is computed by VOSviewer as the sum of the strengths of all links connecting a given item to other items; a higher TLS value indicates stronger overall collaborative or co-occurrence engagement within the network.

## 3 Results

The bibliometric analysis is presented across six sub-sections: publication trends, institutional cooperation, country-level collaboration, author networks, keyword co-occurrence, and journal influence. Each subsection presents the network visualization findings followed by interpretation of the observed patterns.

### 3.1 General trends

In the past few decades, there has been a steady rise in research output related to the effects of mobile phone radiation on the reproductive system. Trends indicate that before 2000, research activity was limited in this area. From 2005 onwards, the publication numbers began to increase, with a significant increase after 2010 (Fig. S1). A peak was observed in research publications between 2018 and 2020, showing increased scientific attention and concern in this domain. Similar observation was seen in citation trends, with a significant rise starting in 2010 and reaching their highest point in 2020 - 2021. The volume of research has not only grown, but its visibility and influence within the academic community have also expanded. As citations generally accumulate gradually over time less citation counts seen in 2024 and 2025 are likely due to the short time span since publication.

### 3.2 Institutional cooperation

An analysis of institutional cooperation reveals a globally distributed yet unevenly connected research landscape. As shown in Table 1, Jawaharlal Nehru University ranking first in both productivity (17 documents) and citations (833) among the institutions analyzed, indicating a substantial contribution to publication output in this domain. Chinese institutions such as Zhejiang University (10 documents and 90 citations) and Shanghai Jiao Tong University (8 documents and 183 citations) also display strong research output. These universities show high total link strength, highlighting their growing global engagement.

**Table 1:** Top 10 Most Productive Organizations by Documents, Citations, and Total Link Strength. TLS as calculated by VOSviewer, represents the sum of the strengths of all co-authorship links of a given institution with other institutions.

Rank	Organization	Documents	Citations	Total Link Strength
1	Jawaharlal Nehru University	17	833	5
2	University of Athens	14	485	6
3	Zhejiang University	10	90	11
4	SuleymanDemirel University	10	389	0
5	Russian Academy of Sciences	9	223	6
6	Karolinska Institute	8	169	13
7	Shanghai Jiao Tong University	8	183	12
8	Université Laval	8	325	8
9	Central South University	8	77	5
10	Islamic Azad University	8	223	4

The network visualization (Fig. 1a) further illustrates distinct institutional clusters, with European universities like the Karolinska Institute (8 documents and 169 citations) and the University of Athens (14 documents and 485 citations) serving as major collaborative hubs that link diverse research groups. In contrast, institutions such as Suleyman Demirel University have contributed a significant number of publications but lack international collaboration, as indicated by their low or absent link strength.

**Fig. 1:**
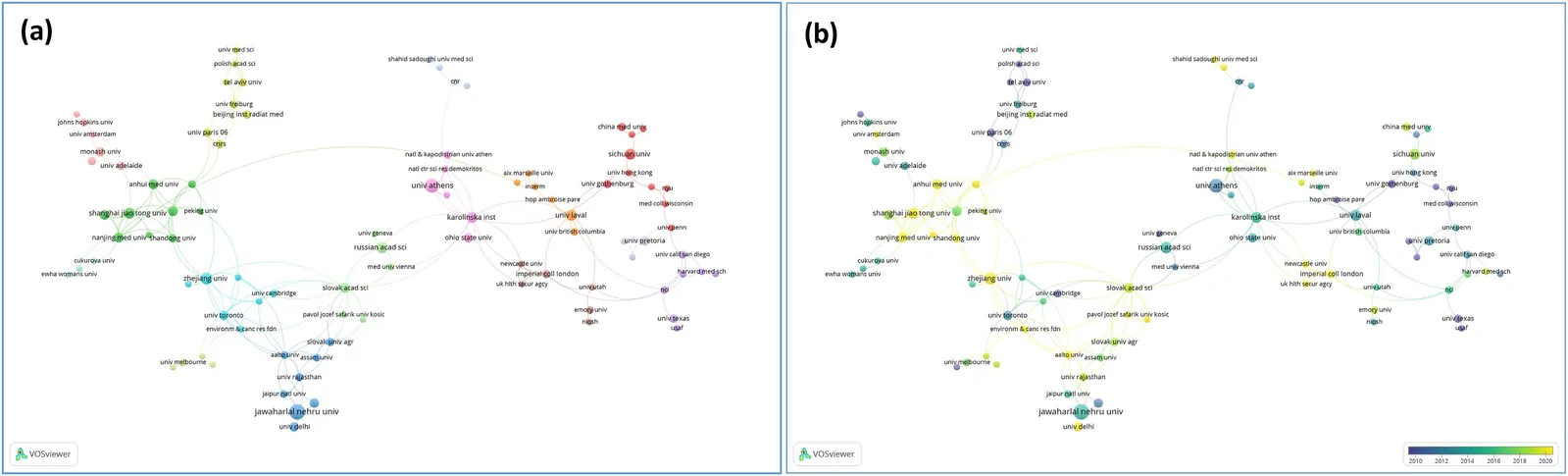
Institutional collaboration network of organizations contributing to RF-EMR and reproductive health research. **(a)** Network visualization: Each node represents an institution; node size reflects the number of publications, and edge thickness indicates the strength of co-authorship links (TLS). Color-coded clusters represent distinct collaborative groups identified by VOSviewer's clustering algorithm. Only institutions with a minimum of three publications and at least one collaborative link are displayed. **(b)** Overlay visualization: Node color reflects the average publication year of each institution, ranging from blue (earlier, ∼2013) to yellow (more recent, ∼2020), illustrating the temporal evolution of institutional research activity.

The overlay visualization (Fig. 1b) reveals a temporal trend, showing that several institutions including Université Laval, Zhejiang University and Jawaharlal Nehru University have intensified their research efforts in recent years (2018–2020), as depicted by yellow-toned nodes. The growing evidence suggests a rising global concern on EMR exposure and its implications on reproductive health.

### 3.3 Countries co-operation

Network visualization reveals that in United States of America and the People's Republic of China have emerged pivotal within the network. The USA, with the highest number of documents (152) and citations (6019), leads in terms of both research output and citations (Table 2), though it should be noted that citation counts are influenced by factors such as publication age, journal visibility, and database coverage rather than research quality alone. Similarly, China with 151 documents and 2218 citations showed a strong volume of research and a strong global linkage, reflecting its significant influence and active participation in international research collaborations. India and Turkey, while slightly less central than the USA and China, are noticeably prominent in the network Fig. 2a and b. Their significant yet growing influence suggests an expanding role in the research community dedicated to studying the impacts of mobile phone radiation. This expansion may be due to increased national funding for research in these areas, and rising national interest due to public health concerns.

**Table 2:** Top 10 Most Productive Countries by Documents, Citations, and Total Link Strength. TLS as calculated by VOSviewer.

Rank	Country	Documents	Citations	Total Link Strength
1	USA	152	6019	103
2	People's Republic of China	151	2218	57
3	India	62	1552	20
4	Turkey	44	1195	14
5	Italy	42	834	36
6	Japan	39	735	7
7	England	37	2202	78
8	Iran	34	643	6
9	Germany	33	1512	54
10	Canada	31	1482	43

TLS, total link strength (see Table 1 for definition).

**Fig. 2:**
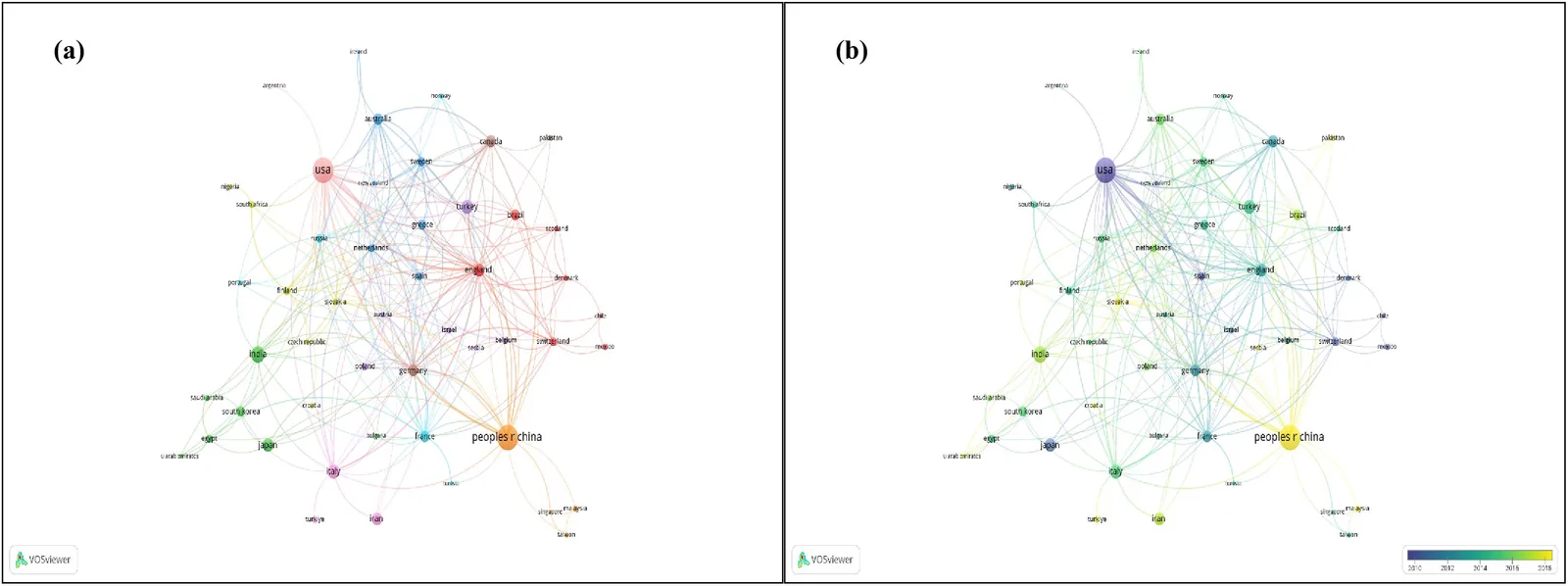
Country-level collaboration network in RF-EMR and reproductive health research. **(a)** Network visualization: Each node represents a country; node size reflects total publication output, and connecting edges represent co-authorship links between countries. TLS indicates the cumulative strength of international collaborations. Only countries with a minimum of five publications are displayed. **(b)** Overlay visualization: Node color indicates the average publication year per country, highlighting temporal shifts in national research contributions.

The visualization highlights significant inter-connectivity between certain nodes, particularly among countries such as the USA, England, Canada, and Germany. Further this inter-connectivity suggesting a robust exchange of knowledge and collaborative ties. Regional clusters, in Europe, where countries like Germany, France, and Italy are closely linked could be indicative of common funding sources, shared research infrastructures, or regional academic or industrial consortium.

The network also illustrates the extensive participation of countries from various continents, highlighting the global concern regarding the health implications of RF-EMF. These country-level patterns represent descriptive bibliometric trends within the Web of Science dataset and should be interpreted with caution, as they may not fully reflect the global research landscape due to database coverage biases and language restrictions.

### 3.4 Authors and cooperation

The author's collaboration analysis (Fig. 3a) reveals distinct clusters of research groups in EMR and reproductive health. Kesari, Kavindra Kumar leads with 10 publications and 685 citations, showing strong centrality (TLS = 22) within an Indian network including Rajamani Paulraj, Behari Jitendra. It should be noted that author-level citation counts and TLS values reflect collaborative visibility within the indexed dataset and are not direct measures of research impact or quality.

**Fig. 3:**
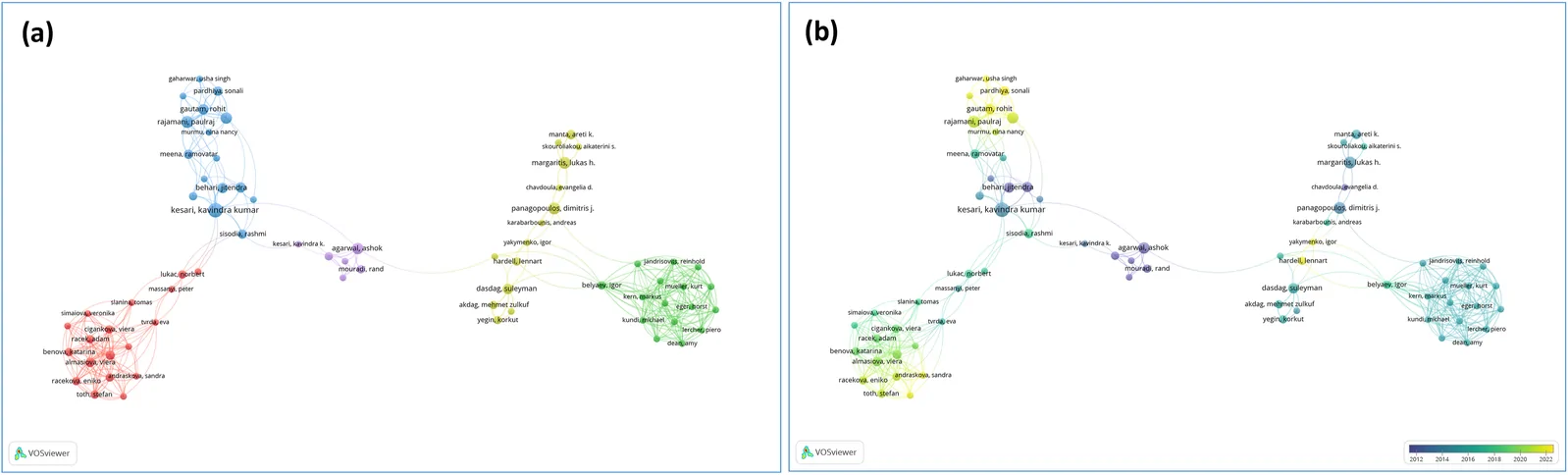
Author co-authorship network in RF-EMR and reproductive health research. **(a)** Network visualization: Each node represents an author; node size reflects total publication count, and edges indicate co-authorship relationships. Color-coded clusters represent distinct collaborative research groups. Only authors with a minimum of three publications are included. **(b)** Overlay visualization: Node color reflects the average publication year of each author's contributions, revealing temporal patterns in individual researcher activity.

Another major group, led by Glueck, C.J. and Wang, P., though less visible in the map, ranks highest in citation impact (716 each) and demonstrates the strongest collaboration (TLS = 26). The red cluster, comprising Lukac, Norbert, Cigankova, Viera, and Racek, Adam, represents a European group with dense internal ties but limited external links. The green cluster, including Belyaev, Igor, Jandrisovits, Reinhold, and Mueller, Kurt, shows strong interconnections with moderate citation impact. Panagopoulos, Dimitris J. and Margaritis, Lukas H. (yellow cluster) have mid-level productivity (7 publications each; 324 and 220 citations) and maintain collaborations with productive authors such as Hardell, Lennart and Yakymenko, Igor.

The overlay visualization (Fig. 3b) highlights temporal patterns: the Indian cluster led by Kesari and Rajamani remains active in 2020–2022, while European groups (e.g., Belyaev, Mueller) show earlier peaks (2014–2018). This indicates a recent shift towards Asian contributions and growing interest in 5G and millimeter-wave effects. Table 3 summarizes the top 10 productive authors, underscoring the role of collaborative networks in shaping future research.

**Table 3:** Top 10 most productive authors in the field of electromagnetic radiation and health research, ranked by number of documents, citations, and total link strength. TLS as calculated by VOSviewer.

Rank	Author	Documents	Citations	Total Link Strength
1	Kesari, Kavindra Kumar	10	685	22
2	Glueck, CJ	8	716	26
3	Wang, P	8	716	26
4	Rajamani, Paulraj	7	133	26
5	Margaritis, Lukas H.	7	220	12
6	Panagopoulos, Dimitris J.	7	324	8
7	Nirala, Jay Prakash	6	52	21
8	Ibrahim, Siti Fatimah	6	52	19
9	Jaffar, Farah HananFathihah	6	52	19
10	Osman, Khairul	6	52	19

TLS, total link strength (see Table 1 for definition).

### 3.5 Analysis of keywords and co-occurrence of keywords

The keyword co-occurrence analysis (Fig. 4a) reveals the major thematic clusters in EMR-related reproductive research.

⁃ The green cluster, dominated by “oxidative stress” (110 occurrences; TLS = 838), highlights it as a central mechanism of EMR-induced reproductive toxicity. Closely linked terms such as “apoptosis”, “sperm”, “semen quality”, and “radiofrequency radiation” emphasize pathways involving oxidative stress-mediated germ cell damage.
⁃ The yellow cluster, featuring “mobile phone radiation” and “microwave radiation”, reflects technological sources of EMR exposure, often associated with sperm motility, DNA fragmentation, and testicular dysfunction.
⁃ The red cluster includes reproductive and hormonal terms such as “testosterone”, “infertility”, “testis”, and “spermatogenesis”, indicating hormonal and cellular outcomes of EMR exposure.
⁃ A distinct blue cluster highlights female reproductive health, with terms like “women”, “pregnancy”, and “polycystic ovary syndrome”, is a significantly understudied area compared to male fertility.

The overlay visualization (Fig. 4b) demonstrates a temporal shift. Recent studies (2011–2018) focus on “mobile phone radiation”, “oxidative stress”, “apoptosis”, and “sperm motility”, while earlier research (2010–2015) emphasized “mutation”, “risk”, and “polymorphism”. This reflects a transition from broad genetic risk studies to targeted mechanistic investigations.

**Fig. 4:**
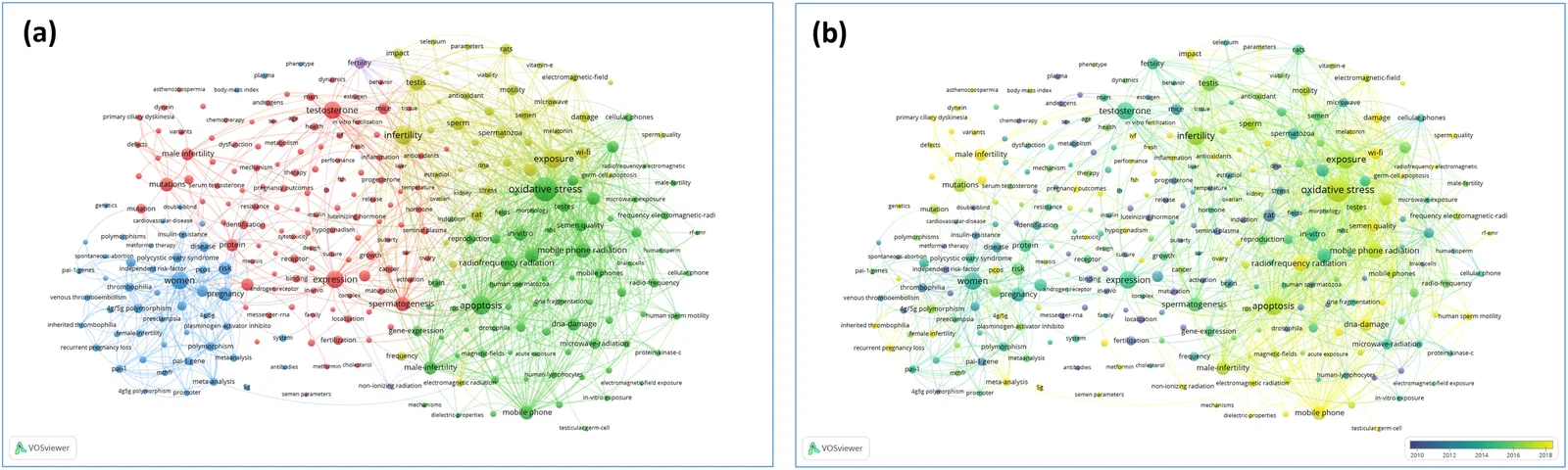
Keyword co-occurrence network derived from 828 publications on RF-EMR and reproductive health. **(a)** Network visualization: Each node represents a keyword; node size reflects occurrence frequency, and edges represent co-occurrence relationships between keywords. TLS indicates the cumulative strength of co-occurrence links. Color-coded clusters represent thematically related keyword groups. Only keywords appearing in five or more publications are displayed. **(b)** Overlay visualization: Node color reflects the average publication year associated with each keyword, illustrating the temporal evolution of research themes from earlier (blue) to more recent (yellow) periods.

In Table 4 the top 20 keywords are summarized. Oxidative stress and apoptosis have emerged as dominant themes overall, linking experimental (in-vitro and animal studies) with EMR exposure. In most studies male infertility remains the primary focus, while pregnancy complications, ovarian dysfunction and hormonal disruptions are under-represented. Notably, 5G radiations is marginally addressed, highlighting the need for future studies on high-frequency RF-EMR, epigenetic alterations, and molecular mechanisms of DNA damage and repair.

**Table 4:** Top 20 keywords by occurrence and total link strength, representing central research themes in EMR and reproductive health. TLS as calculated by VOSviewer.

Keyword	Occurrences	Total Link Strength
Oxidative stress	110	838
Exposure	68	483
Infertility	68	410
Apoptosis	67	534
Testosterone	59	283
Expression	58	272
Women	57	271
Testis	46	305
Mobile phone radiation	44	373
Spermatogenesis	40	232
Radiofrequency radiation	39	315
Pregnancy	38	188
Mobile phone	37	324
Sperm motility	37	280
Mutations	37	123
In-vitro	36	223
Male-infertility	35	290
Sperm	35	203
Risk	32	152
Cell phone	31	271

TLS, total link strength (see Table 1 for definition).

### 3.6 Most relevance sources/journals or analysis of source journals

An analysis of most cited journals by co-citation network (Fig. 5a) and Table 5, shows the publishing landscape on EMR and reproductive health. Bioelectromagnetics is the leading journal, with 16 publications, 728 citations, and a TLS of 77. Electromagnetic Biology and Medicine, with fewer publications (12), records the highest TLS (106), underscoring its central role in inter-journal connectivity. Andrologia (15 publications) and the Journal of Assisted Reproduction and Genetics (13) also contribute significantly, particularly in reproductive toxicology and male fertility. In Fig. 5a, the green cluster, dominated by bioelectromagnetics and environmental health journals, forms the research core, whereas clinical outlets such as clinical endocrinology and human reproduction show moderate impact but weak integration (TLS < 5) (see Table 6).

**Fig. 5:**
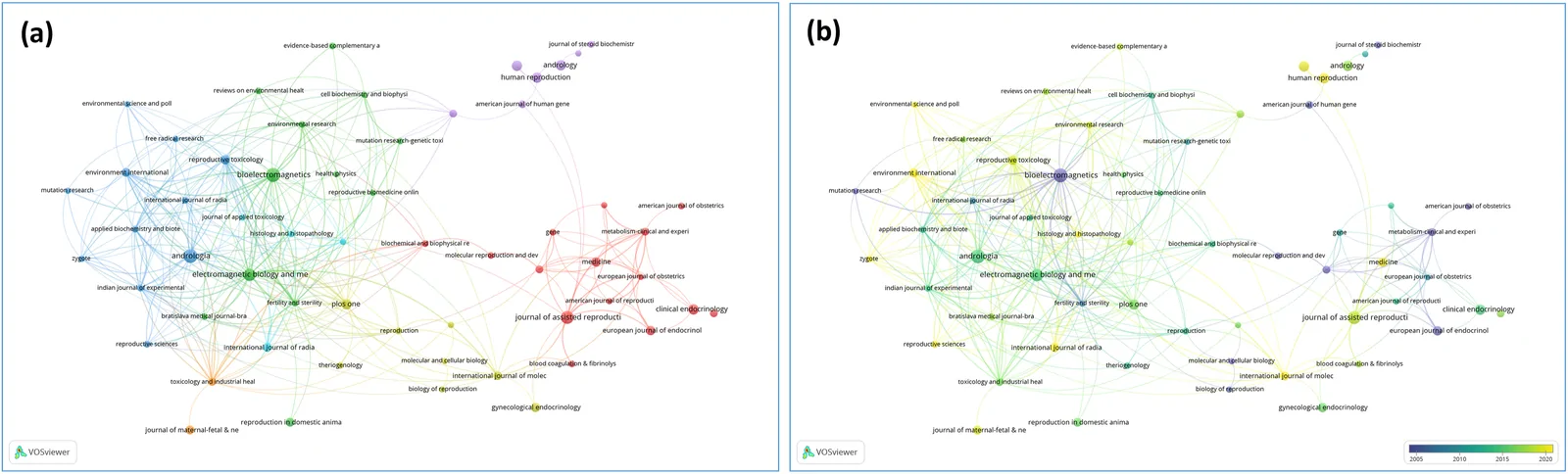
Journal co-citation network identifying core and peripheral journals in RF-EMR and reproductive health research. **(a)** Network visualization: Each node represents a journal; node size reflects the number of publications in the retrieved dataset, and edges indicate co-citation relationships between journals. TLS reflects the cumulative strength of co-citation links. Only journals with a minimum of five publications are displayed. **(b)** Overlay visualization: Node color reflects the average publication year, highlighting temporal shifts in journal prominence within this research domain.

**Table 5:** Top 10 journals contributing to EMR and reproductive health research with corresponding document counts, citations, and total link strength. TLS as calculated by VOSviewer.

Source	Documents	Citations	Total Link Strength
Bioelectromagnetics	16	728	77
Andrologia	15	377	43
Journal of Assisted Reproduction and Genetics	13	216	34
Electromagnetic Biology and Medicine	12	399	106
PLOS ONE	9	376	48
Clinical Endocrinology	9	137	5
Human Reproduction	9	199	3
Andrology	9	201	2
Clinical Genetics	9	137	2
Biotechnology and Bioengineering	9	366	0

TLS, total link strength (see Table 1 for definition).

**Table 6:** Summary of mechanistic evidence linking RF-EMR exposure to reproductive toxicity.

Mechanism	Primary evidence type	Key findings	Keyword co-occurrence	Limitation
Oxidative stress	In vivo (rodent), in vitro	Elevated MDA, reduced SOD, increased ROS	110 occurrences; TLS = 838	Limited human data
Mitochondrial dysfunction	In vitro, in vivo	Reduced MMP, Complex III dysfunction	Co-occurs with oxidative stress	No human direct evidence
DNA damage	In vitro, in vivo, human semen	Increased DFI, 8-OHdG, comet tail length	Co-occurs with sperm, apoptosis	Heterogeneous exposure parameters
Apoptosis	In vivo, in vitro	Caspase-3 activation, Bcl-2 reduction	Co-occurs with oxidative stress	Mostly animal models
Hormonal disruption	In vivo, human epidemiology	Reduced testosterone, LH, FSH	Co-occurs with testosterone, testis	Inconsistent findings
Impaired spermatogenesis	In vivo, human semen analysis	Reduced count, motility, morphology	Co-occurs with sperm motility, count	Confounders not always controlled

Evidence is categorized by study type and key outcome measures. Abbreviations: MDA, malondialdehyde; SOD, superoxide dismutase; ROS, reactive oxygen species; MMP, mitochondrial membrane potential; DFI, DNA fragmentation index; 8-OHdG, 8-hydroxy-2′-deoxyguanosine; LH, luteinizing hormone; FSH, follicle-stimulating hormone; RF-EMR, radiofrequency electromagnetic radiation.

The overlay map (Fig. 5b) shows a temporal shift in the earlier work (pre-2012) which appeared in bioelectromagnetics, mutation research, and reproductive toxicology, while recent studies (2016-2020) are increasingly being published in interdisciplinary journals like PLOS ONE and Environmental Research. Publication counts and TLS values for journals reflect their representation within the retrieved dataset and the co-citation network, and should not be interpreted as absolute measures of journal quality or influence.

Most research is being focused on male fertility, while ovarian and pregnancy related studies are under represented. To study the long term effect of daily EMF exposure from smart phones and 5G there is a need of translational research wherein there is collaborations across the fields of toxicology and endocrinology.

The keyword co-occurrence analysis identified oxidative stress (110 occurrences; TLS = 838), apoptosis, DNA fragmentation, sperm motility, testosterone, and spermatogenesis as the dominant mechanistic themes in the retrieved literature. The following subsections explore the experimental and clinical evidence underlying these themes, organized by mechanistic pathway. Evidence is drawn from in vitro cell studies, in vivo animal models, human semen analyses, and epidemiological studies; these evidence levels are distinguished throughout, as findings from animal and in vitro models cannot be directly extrapolated to human reproductive outcomes without appropriate caution.

## 4 Discussion

We are exposed to different types of ionizing and non-ionizing radiations and both have adverse effects on male and female reproductive system [29]. Research has shown that RF-EMR exposure could have a harmful impact on pregnancy rate, women reproductive health and sperm parameters in experimental mammals [[30], [31], [32]].

The literature has shown that oxidative stress or generation of free radical is one of the main causes behind effects of radiation on biological system [33]. When excessive amounts of reactive oxygen species (ROS) are produced or decline in antioxidant status, the equilibrium between oxidation and reduction is disrupted, causing oxidative stress (OS). As OS increases it affects sperm viability, membrane integrity and fertilization capacity (competence for fusion with the oocyte), which affects fertility and disrupted offspring development [34,35]. At lower levels of oxidative stress, spermatozoa can retain their fertilization capacity even when nuclear DNA has sustained oxidative damage [36].

The institutional collaboration network highlights strong regional research clusters with limited global integration. Despite substantial collaboration made by China, Europe, North America, and India, international research partnerships remain fragmented. The increasing global concern has made it imperative to explore further the potential impact of EMF on reproductive health. Studies undertaken in future should focus on different populations groups to assess genetic, environmental, and lifestyle variations to RF-EMR exposure. Therefore, to have a holistic understanding of RF-EMR effects on reproductive health it is essential to strengthen global collaboration, standardize methodology, and foster inter-disciplinary research.

### 4.1 Mechanistic insights into RF-EMR induced reproductive toxicity

The keyword co-occurrence analysis identified oxidative stress (110 occurrences; TLS = 838), apoptosis, DNA fragmentation, sperm motility, testosterone, and spermatogenesis as the dominant mechanistic themes in the retrieved literature. The following subsections explore the experimental and clinical evidence underlying these themes, organized by mechanistic pathway. Evidence is drawn from in vitro cell studies, in vivo animal models, human semen analyses, and epidemiological studies; these evidence levels are distinguished throughout, as findings from animal and in vitro models cannot be directly extrapolated to human reproductive outcomes without appropriate caution [37,38].

### 4.2 Exposure characteristics and biophysical parameters

The biological effects of radiofrequency electromagnetic radiation on reproductive tissues are influenced by several biophysical and exposure-related parameters, including frequency, specific absorption rate (SAR), power density, exposure duration, distance from the radiation source, tissue conductivity, and dielectric properties [39]. SAR is one of the most widely used parameters for estimating the rate at which electromagnetic energy is absorbed by biological tissues. It is expressed in watts per kilogram (W/kg) and depends on tissue conductivity, electric field strength, and tissue density. Variations in SAR values among studies may significantly influence biological outcomes and contribute to inconsistencies in experimental findings [40]. Further a specific RF-EMR exposure level for maintaining optimal sperm function has not yet been established. The ICNIRP 2020 guidelines set the maximum whole-body SAR for the general public at 0.08 W/kg and the maximum local SAR for the head and torso at 2 W/kg averaged over 10 g of tissue. These limits are for human protection and should not be considered as a threshold for sperm cells [41].

Besides SAR, modulation pattern, polarization, exposure intermittency and proximity of the device to reproductive organs are few factors which also determine degree of biological interaction [42]. The differences in exposure conditions between in vitro, in vivo and epidemiological study is a challenge to establish standardized conclusions on reproductive toxicity due to RF-EMR exposure. In addition to SAR, factors such as modulation pattern, polarization, exposure intermittency, and proximity of the device to reproductive organs also determine the extent of biological interaction. RF-EMR may exert both thermal and non-thermal effects.

Thermal effects arise due to tissue heating caused by energy absorption, whereas non-thermal effects are believed to involve alterations in cellular signaling pathways, oxidative balance, membrane permeability, and mitochondrial function without significant temperature elevation [40]. The diversity in exposure conditions across in vitro, in vivo, and epidemiological studies remains a major challenge in establishing standardized conclusions regarding reproductive toxicity associated with RF-EMR exposure.

### 4.3 Oxidative stress as a central mechanism of RF-EMR toxicity

Oxidative stress is one of the most widely proposed mechanisms responsible for RF- EMR induced reproductive toxicity [43]. Experimental studies have shown that RF-EMR exposure can increase the reactive oxygen species (ROS) generation and thus affects the redox homeostasis in the reproductive tissues [44]. The majority of evidence linking RF-EMR exposure to oxidative stress in reproductive tissues comes from in vivo rodent studies and in vitro cell line experiments, with a smaller body of human semen studies providing supporting clinical data. Excessive ROS production may injure cellular macromolecule including lipids, protein and nucleic acid, eventually impairing reproductive function [45]. In animal and in vitro models, RF-EMR exposure is often associated with increased malondialdehyde (MDA) levels, reduced antioxidant enzyme activity, altered glutathione balance and increased lipid peroxidation [8,46]. The prominence of oxidative stress in the keyword co-occurrence network (110 occurrences; TLS = 838) reflects the central role this mechanism occupies in the published literature on RF-EMR and reproductive toxicity.

#### 4.3.1 ROS generation through fenton-type reactions

One of the suggested mechanisms for RF-EMR induced oxidative stress is the stimulation of metal ion mediated oxidative reactions (Fenton Reaction) [47]. Transition metals such as iron and copper can catalyse the conversion of hydrogen peroxide to highly reactive hydroxyl radicals in biological systems. RF-EMR exposure indirectly promotes these reactions via disturbing the intracellular ionic balance and increasing the generation of free radicals [23]. This increase in hydroxyl radical production can lead to lipid peroxidation, protein oxidation and DNA damage [45]. Although direct evidence for RF-EMR exposure causing classical Fenton reactions is lacking, some data support the hypothesis that RF-EMR may facilitate oxidative pathways of transition metal-dependent ROS generation. Further mechanistic studies are needed to understand the exact molecular interactions.

#### 4.3.2 Mitochondrial dysfunction and NADH oxidase activation

Mitochondria are considered one of the primary intracellular targets of RF-EMR exposure. Experimental studies suggest that RF-EMR can disrupt mitochondrial membrane potential and electron transport chain activity, resulting in electron leakage and excessive superoxide radical formation [48]. In addition to direct mitochondrial damage, RF-EMR-induced metabolic stress may engage AMPK-dependent cellular protection pathways as a compensatory response to bioenergetic disruption; however, chronic or high-intensity exposure may overwhelm these adaptive mechanisms, ultimately leading to mitochondrial dysfunction and impaired spermatogenesis [49]. Furthermore, ROS-dependent alterations in mitochondrial dynamics have been shown to trigger autophagic responses as a cellular adaptation to oxidative injury; while transient autophagy may serve a protective role, sustained autophagic flux under chronic RF-EMR exposure may contribute to germ cell loss and testicular dysfunction [50]. Altered mitochondrial function may impair ATP synthesis and enhance oxidative stress within reproductive cells. Additionally, activation of membrane-associated NADH oxidase has been proposed as another source of RF-EMR-induced ROS production. Increased NADH oxidase activity may stimulate the generation of superoxide anions, further amplifying oxidative damage [51,52]. Since spermatozoa rely heavily on mitochondrial ATP production for motility and fertilization capacity, mitochondrial dysfunction induced by RF-EMR may significantly contribute to impaired sperm function and reduced fertility potential [53,54]. Recent evidence further highlights that disruption of mitochondrial dynamics including imbalances between mitochondrial fusion and fission may serve as a key mechanistic link between environmental stressor exposure and tissue-level injury, reinforcing the relevance of mitochondrial homeostasis as a target in RF-EMR-induced reproductive toxicity [55].

### 4.4 Oxidative stress and sperm dysfunction

Physiological levels of ROS are essential for normal sperm function, including capacitation, hyperactivation, acrosome reaction, and sperm-oocyte fusion [56]. However, excessive ROS generation results in oxidative stress, which adversely affects sperm quality and reproductive potential. Spermatozoa are particularly vulnerable to oxidative damage due to their limited cytoplasmic content and comparatively weak antioxidant defense system [57]. Oxidative stress-mediated loss of mitochondrial membrane potential and activation of PARP-1-associated injury pathways have been identified as critical downstream events linking ROS accumulation to cell death in reproductive tissues, providing a mechanistic bridge between RF-EMR-induced redox imbalance and germ cell apoptosis [58].

Additionally the recent evidence further supports the association between RF-EMR exposure, oxidative stress and male reproductive outcomes. Experimental evidence also indicates potential alterations in oxidative-stress biomarkers in testicular tissue, although the overall certainty of evidence remains low [59]. More recent animal studies on reproductive biomarkers also emphasize the significance of antioxidant status and redox balance in maintaining reproductive function [60]. Human observational evidence suggests that the effects of RF-EMR exposure on sperm concentration, morphology, motility and total sperm count remain inconclusive, mainly due to limitations in exposure assessment and study design [61].

Moreover, the sperm plasma membrane is highly enriched with polyunsaturated fatty acids (PUFAs), making it highly susceptible to lipid peroxidation. Oxidative damage to membrane lipids can reduce membrane fluidity, impair sperm motility, and compromise membrane integrity [62]. RF-EMR-induced oxidative stress has been associated with decreased sperm concentration, reduced motility, abnormal morphology, mitochondrial dysfunction, and increased DNA fragmentation in several experimental and clinical studies. In addition to affecting sperm parameters directly, oxidative stress may also alter seminal plasma antioxidant capacity and disrupt the redox balance necessary for maintaining sperm viability and fertilization competence [[63], [64], [65], [66]]. These findings collectively suggest that oxidative stress serves as a major mediator linking RF-EMR exposure to male reproductive dysfunction.

### 4.5 Effects on hormonal and endocrine parameters

Evidence for RF-EMR-induced hormonal disruption and impaired spermatogenesis spans in vivo animal studies, human semen analyses, and a limited number of epidemiological studies, though findings remain inconsistent across exposure parameters. RF-EMR exposure may also influence reproductive health through disruption of endocrine and hormonal regulation. Several animal and human studies have reported alterations in serum testosterone, luteinizing hormone, and follicle-stimulating hormone levels following prolonged RF-EMR exposure. Such hormonal disturbances may impair spermatogenesis and testicular function. Leydig cells, responsible for testosterone synthesis, appear particularly susceptible to oxidative stress-induced dysfunction [67]. Reduced testosterone production may subsequently affect sperm maturation, libido, and fertility potential. Similarly, RF-EMR-induced oxidative damage in Sertoli cells may compromise nutritional and structural support required for germ cell development. However, findings regarding hormonal alterations remain inconsistent across studies, likely due to differences in exposure conditions, duration, species variability, and experimental design. Further well-standardized investigations are required to establish the long-term endocrine consequences of chronic RF-EMR exposure. The keyword co-occurrence of 'testosterone', 'spermatogenesis', 'testis', and 'Leydig cells' within the red thematic cluster of the bibliometric map is consistent with the hormonal and cellular endpoints described in this section.

### 4.6 DNA damage and genotoxic effects

Although numerous in vitro and in vivo studies have demonstrated that radiofrequency electromagnetic radiation (RF-EMR) can induce sperm DNA damage, epidemiological evidence in human populations remains limited and is characterized by considerable methodological heterogeneity. Current evidence suggests that RF-EMR may exert genotoxic effects on reproductive cells primarily through oxidative stress-mediated mechanisms. Excessive production of reactive oxygen species (ROS) following RF-EMR exposure can overwhelm endogenous antioxidant defenses, resulting in oxidative damage to nucleic acids, DNA strand breaks, chromosomal instability, and oxidative base modifications [68]. Experimental studies have consistently reported increased sperm DNA fragmentation and compromised chromatin integrity following prolonged RF-EMR exposure [69,70]. Because sperm DNA integrity is essential for successful fertilization, normal embryonic development, and healthy offspring, such alterations may have important implications for male reproductive health [71]. Furthermore, comet assay analyses have demonstrated significant increases in tail length and tail moment in RF-EMR-exposed germ cells, indicating enhanced DNA strand damage. Consistent with these findings, elevated levels of 8-hydroxy-2′-deoxyguanosine (8-OHdG), a well-established biomarker of oxidative DNA damage, have also been detected in reproductive tissues following RF-EMR exposure, further supporting oxidative stress as a central mechanism underlying RF-EMR-induced genotoxicity [72].

### 4.7 Effect on female reproductive system

In contrast to male-centric data, the impact of RF-EMR on female reproductive health is less studied. However recent animals studies have suggested that ovarian tissues may be more prone to RF-EMR induced oxidative stress [3]. Studies have also shown that RF-EMR exposure could cause increased ROS production, lipid peroxidation and mitochondrial dysfunction in ovarian tissue [48]. This will further lead to follicular degeneration, oocyte maturation failure and decline in reproductive capacity. Concurrently RF-EMR can also affect endocrine regulation via changes in the hypothalamic-pituitary-ovarian axis. Animal studies also reported that on exposure to electromagnetic field changes in hormonal levels (estrogen, progesterone, FSH and LH), increase in oxidative stress and alterations in uterine histopathology were observed [73,74]. Corroborating these findings highlighted that the results are not consistent across exposure conditions and experimental models. Recent study has also raised concern regarding potential effects of RF-EMF on pregnancy and birth outcomes. A systematic review on human observational studies showed limited but for associations between RF-EMR exposure and adverse female reproductive outcomes [10]. Authors also highlighted methodological heterogeneity and need for improved exposure assessment. Notably, a cohort study conducted among pregnant women suggested associations between electromagnetic wave exposure and certain adverse pregnancy and birth outcomes, although causal relationships remain uncertain [31]. Recent experimental evidence has further highlighted potential effects of emerging wireless technologies on female reproductive system. Exposure of female Wistar rats to 700 MHz (5G lower band frequency) caused significant changes in oxidative stress biomarkers and reproductive hormones [27].

### 4.8 Emerging concerns related to 5G and millimeter-wave exposure

The rapid global deployment of fifth-generation (5G) wireless technology has raised new concerns about potential biological and reproductive health effects associated with high-frequency RF-EMR exposure. 5G technology operates on higher frequency bands such as millimeter waves and it relies on dense network infrastructure [75]. However long term biological effects of millimeter waves were poorly understood despite their less tissue penetration than lower frequency RF-EMR. Existing reproductive toxicity studies have primarily focused on frequencies associated with 2G, 3G, and 4G technologies, while experimental evidence related to 5G exposure is limited. So the increasing use of wireless communication technologies emphasizes the urgent need for well standardized studies to assess the reproductive and systemic effects of chronic low-level 5G exposure.

## 5 Limitation of the study

The inclusion of only English-language publications introduces a language bias. Additionally, the search strategy and chosen keywords may have missed relevant articles. Citation counts, used to assess impact, can be influenced by self-citations and do not always reflect research quality. Recent publications may also be under represented due to indexing delays. Lastly, articles were included from only one database (Web of Science) which may have limited the comprehensiveness of the review.

Several limitations of bibliometric indicators must be acknowledged.

⁃ Publication counts reflect research output volume but not research quality or methodological rigor.
⁃ Citation counts are influenced by publication age, self-citation practices, journal visibility, open-access status, and field size, and should not be interpreted as direct measures of scientific impact.
⁃ Total link strength (TLS) reflects collaborative connectivity within the indexed dataset and does not capture informal collaborations or inter-disciplinary contributions that fall outside the search parameters.
⁃ Country and institutional prominence in network maps reflects patterns within the Web of Science dataset only and may not represent the true global research landscape, particularly for research published in non-English languages or indexed in other databases.

## 6 Conclusion

This bibliometric analysis provides valuable insights into the research on RF-EMR emitted from mobile phones and its effects on the reproductive health. The study identified major trends, key collaborations, and central theme focuses. The network visualization of keywords demonstrates that research in this domain is primarily centered around oxidative stress, apoptosis, sperm quality, testosterone, and infertility. Recent studies have shown a growing interest towards understanding the cellular and molecular impact of RF-EMF.

In the coming years research should focus on multi-centric studies, long-term exposure assessments, and interdisciplinary approaches, incorporating epidemiology, toxicology, and computational modeling. Due to limited collaboration between clinical researchers and experimental biologists in previous studies, it is essential to have integrative studies. As technology continues to evolve, newer research methodologies will be required to understand the long-term implications of electromagnetic radiation on human health.

## CRediT authorship contribution statement

**Rohit Gautam:** Writing – original draft, Methodology, Conceptualization. **Anuj Kumar Tomar:** Writing – original draft, Data curation. **Shweta Tanwar:** Writing – review & editing. **Poulami Mazumder:** Writing – original draft, Conceptualization. **Anita Sharma:** Writing – review & editing. **Asha Verma:** Writing – review & editing, Conceptualization. **Taruna Arora:** Writing – review & editing, Supervision, Conceptualization.

## Institutional review board statement/ethical statement

Not Applicable.

## Generative AI statement

The author(s) declare that Generative AI was used in the creation of this manuscript. Artificial intelligence (ChatGPT) assistance was used to improve and correct English language (grammar and spelling).

## Funding

This work was supported by the Indian Council of Medical Research (ICMR) under Grant ID 5/10/NTF/FR/32/2020-RBMCH

## Declaration of competing interest

The authors declare that they have no known competing financial interests or personal relationships that could have appeared to influence the work reported in this paper.

## Data Availability

Data will be made available on request.
