# Supplementary material for: Global research trends on RF-EMR and reproductive health: Bibliometric insights and mechanistic review
Source: Biochem Biophys Rep. 2026 Oct 9;48:102832. doi: 10.1016/j.bbrep.2026.102832 (PMC13677566; doi:10.1016/j.bbrep.2026.102832)
Supplement: Supplementary file 1 [file mmc1.docx]

**Figure S1:** Annual publication and citation trends in RF-EMR and reproductive

health research (retrieved from Web of Science, up to February 2025). Bar

graph showing the number of publications per year and cumulative citations

per year.

**Table S1. Search strategy**

| **No.** | **Web of Science Search Key terms** | **Results**  **(as of 19/02/2025)** |
| --- | --- | --- |
| #1 | **TS=("Mobile Phone Radiation" OR "Cell Phone Radiation" OR "Microwave Radiation" OR "Radio Frequency Radiation" OR "Non-Ionizing Radiation" OR "RFR" OR "1G" OR "2G" OR "3G" OR "4G" OR "5G" OR "6G" OR "Wi-Fi" OR "2.45GHz" OR "Mobile Phone Tower" OR "Cell Phone Tower" OR "Telecommunication Tower")** | [**115470**](https://www-webofscience-com-aiims.knimbus.com/wos/woscc/summary/37c5eccb-3288-4e74-ba5d-e00441cd6046-01546a7f3c/relevance/1) |
| #2 | **TS=(“Infertility” OR “Male infertility” OR “Male Reproductive System” OR “Semen Quality” OR “Sperm” OR "Sperm Abnormalities" OR “Spermatogenesis” OR "Semen Parameters" OR “Testosterone” OR "Male Reproductive Hormone" OR “Spermatozoa" OR "Sperm Motility" OR "Sperm Count" OR "Sperm Morphology" OR "Sperm Chromatin" OR "Sperm DNA Fragmentation" OR "Epididymis" OR“Testis” OR "Leydig Cells" OR "Sertoli Cells" OR "Testicular Dysfunction" OR “Female Reproductive System” OR “Ovary” OR “Ovary Function” OR “Ovarian Reserve” OR “Pregnancy Outcomes” OR “Oogenesis” OR “FSH” OR “Follicle Stimulating Hormone” OR “LH” OR “Luteinizing Hormone”** | **590,991** |
| #3 | Combine #1 AND #2 | **877** |
|  | Filters applied: |  |
|  | **#1 AND #2 and Article or Review Article (Document Types) and English (Languages)** | **828** |
